# Novel preparation of PLGA/HP55 nanoparticles for oral insulin delivery

**DOI:** 10.1186/1556-276X-7-299

**Published:** 2012-06-08

**Authors:** Zhi Min Wu, Li Ling, Li Ying Zhou, Xin Dong Guo, Wei Jiang, Yu Qian, Kathy Qian Luo, Li Juan Zhang

**Affiliations:** 1 School of Chemistry and Chemical Engineering, South China University of Technology, Guangzhou, 510640, People’s Republic of China; 2 Department of Chemical and Bio-molecular Engineering, The Hong Kong University of Science and Technology, Clear Water Bay, Kowloon, Hong Kong; 3 Division of Bioengineering, School of Chemical and Biomedical Engineering, Nanyang Technological University, 70 Nanyang Drive, 637457, Singapore

**Keywords:** pH-sensitive nanoparticles, multiple emulsions, oral insulin, diabetes

## Abstract

The aim of the present study was to develop the PLGA/HP55 nanoparticles with improved hypoglycemic effect for oral insulin delivery. The insulin-loaded PLGA/HP55 nanoparticles were produced by a modified multiple emulsion solvent evaporation method. The physicochemical characteristics, *in vitro* release of insulin, and *in vivo* efficacy in diabetic rats of the nanoparticles were evaluated. The insulin encapsulation efficiency was up to 94%, and insulin was released in a pH-dependent manner under simulated gastrointestinal conditions. When administered orally (50 IU/kg) to diabetic rats, the nanoparticles can decrease rapidly the blood glucose level with a maximal effect between 1 and 8 h. The relative bioavailability compared with subcutaneous injection (5 IU/kg) in diabetic rats was 11.3% ± 1.05%. This effect may be explained by the fast release of insulin in the upper intestine, where it is better absorbed by the high gradient concentration of insulin than other regions. These results show that the PLGA/HP55 nanoparticles developed in the study might be employed as a potential method for oral insulin delivery.

## Background

Insulin is the most effective medicine in lowering the glucose level of blood for the treatment of diabetes mellitus [1]. Early introduction of insulin can also protect islets from apoptosis and increase β-cell regeneration in type 2 diabetes [2]. Subcutaneous injections of insulin remain to be the preferred approach for diabetic patients but often result in poor patient compliance [3,4]. Oral administration of insulin seems to be the most convenient way and can mimic endogenous production of insulin [5]. However, a reliable insulin formulation for the oral delivery is encountered with some barriers in the gastrointestinal (GI) tract that include (a) enzymatic degradation in the GI tract and (b) poor insulin permeability through the GI system [6]. The bioavailability of insulin solution delivered orally is less than 1% [7].

pH-sensitive polymers have been developed and introduced into the particulate carriers to circumvent the barriers for oral insulin delivery. As early as 1999, insulin was formulated with pH-sensitive microparticles, and a prolonged reduction of hyperglycemia was observed after oral administration to diabetic rats [7]. This reduction was dose dependent and lasted up to 8 h with a dose of 25 IU/kg of encapsulated insulin. These results were confirmed by pH-responsive hydrogel particles and demonstrated a similar manner in the reduction of blood glucose level after oral administration of polymeric dosage form and direct injection of insulin [8,9]. Generally, nanoparticles have greater intracellular uptake compared with microparticles and are available to a greater range of biologic targets due to their smaller size and mobility [10]. Depending on the pH-sensitive polymer combinations, nanoparticles can be tailored to control release kinetics, facilitate the uptake of insulin, and increase the oral bioavailability [11,12]. Various pH-sensitive nanoparticles based on pH-sensitive polymers were developed for oral delivery of insulin. These pH-sensitive polymers include polymethacrylic acid [13-18], hydroxypropyl methylcellulose phthalate (HPMCP(HP55)) [19], dextran sulphate [20-23], alginate [21,24], poly-γ-glutamic acid [25,26], and so on. Generally, these formulations prepared with these pH-sensitive polymers, orally administered mostly to diabetic rats, induced a different extent reduction in blood glucose level.

However, absorption of insulin from different regions of the intestine is not uniform due to the cellular morphology of the intestines’ changes from region to region [23,27-29]. Interestingly, the plasma insulin concentration-time curves after orally delivered nanoparticles showed different insulin peaks. According to the time of insulin peaks, the ways of insulin absorption could be divided into the fast and the slow absorption way (the dividing point is often observed in 8 h of post-administration). The fast absorption way is probably related to insulin directly absorbed through paracellular pathway [23,24,28,30]. The slow absorption way is possibly related to higher insulin residence time at the absorption site and delay of nanoparticle uptake by endocytosis via enterocytes or M cells in the small intestine [23,24,29]. Insulin within carrier could be directly absorbed through the intestinal wall exerting a hypoglycemic effect [31,32]. Insulin level in blood after intraduodenal injection of insulin/carrier solution showed the insulin peak occurred in 20 to 40 min [33,34]. The upper intestinal area seems to be the most active region for insulin absorption [35]. In addition, nanoparticles orally delivered are an aqueous suspension; they will leave the stomach of fasted animals very rapidly and arrive in the upper intestine where they will be able to release insulin [28]. Hence, the nanoencapsulated insulin rapidly absorbed may occur primarily in the upper intestine.

We hypothesized that the insulin, remaining high concentration due to the fast release from the pH-sensitive nanoparticles with high entrapment efficiency, should be better absorbed by the upper intestinal mucosa and, in consequence, should rapidly produce the biological efficacy. In addition, the rapid adsorption of insulin in the upper intestine would decrease the loss of drug across the intestine. Thus, the present study aims to develop an oral dosage of insulin-loaded nanoparticles composed of poly (lactide-*co*-glycolide (PLGA)/HP55 with strong drug encapsulation ability and good pH-sensitive release property. The nanoparticles were prepared by a novel technique of modified multiple emulsion solvent evaporation (MESE) method. The physicochemical characterization (morphology, size, insulin loading, and *in vitro* release of insulin) of nanoparticles was examined and evaluated. The biological efficacy after oral administration of insulin-loaded PLGA/HP55 nanoparticles in diabetic rats was studied.

## Methods

### Materials

Pure crystalline porcine insulin was purchased from Xuzhou Wanbang Bio-Chemical Co. Ltd., (No. 0312A02, Jiangsu, China), with a nominal activity of 27 IU/mg. PLGA (50/50, Mw is about 20,000) was acquired from Shandong Medical Instrument Institute (Shandong, China). The HPMCP (HP55, Mw is about 45,000) and polyvinyl alcohol (87% hydrolysed, Mw is about 31,000 to 50,000) were purchased from Acros Organics (NJ, USA). All other reagents and solvents used were analytical grade. Streptozotocin (STZ; HPLC) was purchased from Sigma-Aldrich, Corporation, St. Louis, MO, USA. Distilled and deionized water (Mili-Q water systems, Millipore, Bedford, MA, USA) was used for the preparation of all sample solutions.

### Animals

Male sprague Dawley rats weighing 220 to 250 g, 12 to 13 weeks old, were provided by the pharmacological laboratory of the Hong Kong University of Science and Technology. They received standard laboratory chow diet and tap water, available *ad libitum*. All experiments were carried out in accordance with the Guideline for the Care and Use of Experiment Animals in Hong Kong University of Science and Technology, Hong Kong.

### Preparation of insulin-loaded PLGA/HP55 nanoparticles

The preparation of nanoparticles was carried out by the modified MESE technique. Briefly, the inner aqueous phase of pH 2.0 insulin solution (10 mg/mL, 1 mL) was added into the oil phase of polymer solution (PLGA/HP55, 50 mg/100 mg) using methylene chloride and acetone (3 mL/2 mL) and then the resulting mixture was emulsified to form the primary emulsion by sonification at power of 40 W for 0.5 min (SONOPULS, Bandelin, German). The primary emulsion was thereafter poured into 20 mL of external aqueous phase of polyvinyl alcohol solution (1%, *w*/*v*) and sonicated at power of 60 W for 1 min, involving the formation of the multiple emulsions. After evaporation of solvents under reduced pressure, the nanoparticles were collected by centrifugation at 20,000 rpm for 10 min and then washed three times with distilled water. The insulin-loaded PLGA/HP55 nanoparticles were vacuum freeze-dried for 24 h after prefreezing of the resultant dispersion at −20°C overnight.

### Characterization of nanoparticles

The size of nanoparticles was determined by photon correlation spectroscopy at 25°C with a detection angle of 90° using a Malvern Zetasizer II (Malvern Instruments Ltd., Worcestershire, UK). Measurements were made on aqueous dilute nanoparticle suspension.

The morphology of nanoparticles was determined by scanning electron microscopy (SEM) (LEO 1530 VP, LEO Elektronenmikroskopie GmbH, Oberkochen, Germany) and transmission electron microscopy (TEM) (Hitachi JEM-100 CXII, Hitachi Ltd., Chiyoda, Tokyo, Japan). For SEM measurement, the powder of nanoparticles was fixed on an aluminum stub as a thin film and coated with gold before observation. SEM was performed at an accelerating voltage of 10 kV and a magnification of × 20,000 in the transmission electron mode. For TEM measurement, an aqueous droplet of nanoparticle solutions was immobilized on copper grids and negatively stained with phosphotungstate solution (2%, *w*/*v*). After drying at room temperature, the morphology of the nanoparticles was examined.

The drug encapsulation efficiency (EE) and loading capacity (LC) were calculated by the following formulas [36]:

(1) $$\text{EE}\;\left(\%\right)=\frac{\left(M_{\text{total}}-V_{\text{supernatant}}\times C_{\text{supernatant}}\right)}{M_{\text{total}}}\times 100\;\%$$

(2) $$\text{LC}\;\left(\%\right)=\frac{\left(M_{\text{total}}-V_{\text{supernatant}}\times 100\;\%\right)}{M_{\text{total}}}\text{,}$$

where *M*_total_ is initial amount of insulin (mg); *V*_supernatant_, volume of supernatant (mL); *C*_supernatant_, concentration of insulin in supernatant (mg/mL); and *W*_nanoparticle_, weight of nanoparticles.

The concentration of insulin was determined by reverse phase HPLC method (Agilent 1200, ZORBAX 300 SB-C18 column 150 mm × 4.6 mm, 5 μm, Agilent Technologies Inc., Santa Clara, CA, USA).[37] The mobile phase consisted of a premixed isocratic mixture of 0.2 M sodium sulfate anhydrous solution adjusted to pH 2.3 with phosphoric acid and acetonitrile (73:27, *v*/*v*). The injection volume for samples and standards were 20 μL and eluted at a flow rate of 0.8 mL min^−1^ at 30°C. The absorbance of insulin was determined using the UV trace at 214 nm.

### *In vitro* release study

Five milligrams of the insulin-loaded nanoparticles were dispersed in 5-mL simulated gastric fluid (pH 1.2) and simulated intestinal fluid (pH 7.4), respectively, and shaken at 50 rpm at 37.8°C using a constant-temperature shaker (SHA-B, Guohua Co. Ltd., Jiangsu, China). At specified time intervals (0, 30, 60, 120, 180, 240, and 480 min), supernatants were collected by centrifugation. The concentrations of insulin in the supernatant were determined by reverse phase HPLC method, and total amount of insulin released from the nanoparticles was calculated. Each experiment was carried out in triplicate.

### *In vivo* study in diabetic rats

Male sprague Dawley rats were made diabetic prior to the study by intravenous injection of 60-mg/kg STZ dissolved in citrate buffer at pH 4.5. They were considered to be diabetic when the fasted glucose levels exceeded 250 mg/dL 2 weeks after STZ treatment. The rats were then used to evaluate the hypoglycemic effects of the insulin-loaded PLGA/HP55 nanoparticles.

Diabetic rats were fasted for 12 h prior to and remained fasted during the experiment but were allowed free access to water throughout the whole experiment. Blood samples were collected from the tail veins of rats prior to drug administration and at different time intervals after dosing. The blood glucose was then determined by a glucose meter using ACCU-Chek Active (Roche Diagnostics, Mannheim, Germany).

Hypoglycemic effect was evaluated by the decrease of plasma glucose levels relative to the basal values in rats divided in three groups (*n* = 3/group) corresponding to (a) subcutaneous (SC)-injected free-form insulin at dose of 5 IU/kg and (b) orally delivered insulin-loaded nanoparticles and (c) orally delivered blank nanoparticles.

Plasma insulin levels were determined by a solid two-side enzyme immunoassay (ELISA test kit, Mercodia, Uppsala, Sweden) based on the direct sandwich technique in which two monoclonal antibodies are directed against separate antigenic determinants on the inulin molecule. The plasma were separated by centrifugation (3,000 rpm, 4°C, 20 min) and samples were stored at 2°C to 8°C, then 25μL of sample added to each well of a 96-well microplate using the assay’s protocol. The insulin concentration was quantified against a standard curved using absorbance at 450 nm (Wallac VICTOR plate reader 1420, Perkin Elmer, Waltham, MA, USA). Plasma insulin levels were plotted against time to evaluate the relative bioavailability. The relative bioavailability (BA_R_) was obtained according to formula (3).

(3) $${\text{BA}}_{\text{R}}\;\left(\%\right)=\frac{{\text{AUC}}_{\text{oral}}\times {\text{Dose}}_{\text{SC}}}{{\text{AUC}}_{\text{SC}}\times {\text{Dose}}_{\text{oral}}}\times 100\;\%\text{,}$$

where AUC_oral_ is the total area under the serum insulin concentration vs. time curve of oral administration of insulin nanoparticles; AUC_SC_, total area under the serum insulin concentration vs. time curve of pure insulin injection; Dose_SC_, dosage of pure insulin injection (IU/kg); and Dose_oral_, dosage of oral administration of insulin nanoparticles (IU/kg).

### Statistical analysis

Data are presented as mean ± standard deviation (S.D.). Comparison between two groups was analyzed by the one-tailed Student’s *t*-test. A statistical difference was considered when *p* value was less than 0.05.

## Results and discussion

### Preparation and characterization of insulin-loaded PLGA/HP55 nanoparticles

**Figure 1:**
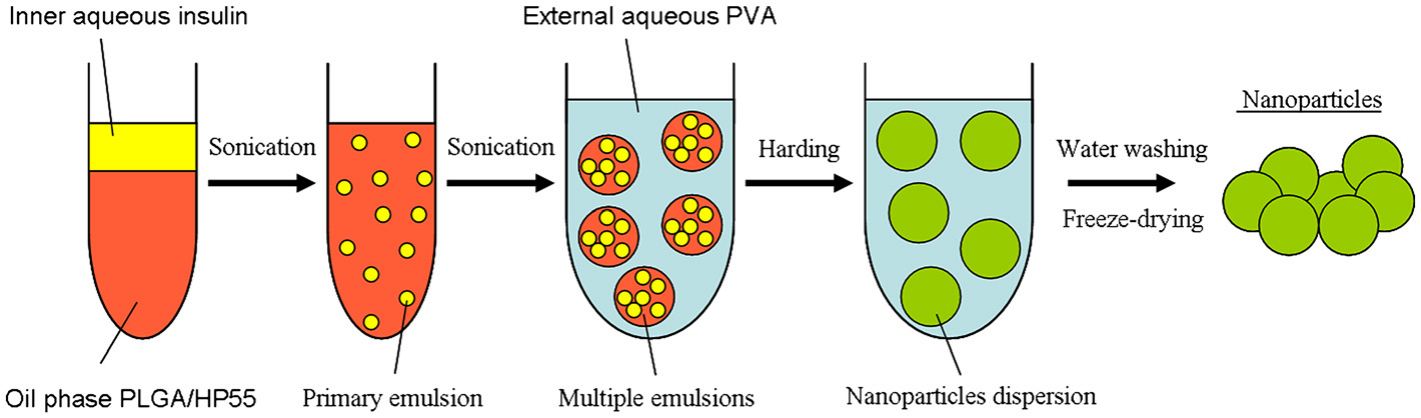
Scheme of the preparation of insulin-loaded PLGA/HP55 nanoparticles.

Polymeric nanoparticles based on the HP55 could not be produced by the traditional multiple emulsion solvent evaporation method due to the poor dissolution of HP55 in a solvent (such as methylene chloride or ethyl acetate), which can easily be removed by evaporation or by the other extraction. In general, the hydrophilic molecules such as insulin can be encapsulated in water-containing emulsion. Although the PLGA/HP55 nanoparticles were prepared by single emulsion solvent diffusion (SESD) method in the literature, using the acetone/water as the solvent, the encapsulation efficiency as well as pH-sensitivity is poor [19]. These properties in PLGA/HP55 nanoparticles could significantly contribute to the low absorption of insulin in the intestine. As shown in Figure 1, novel insulin-loaded PLGA/HP55 nanoparticles were developed by the modified MESE method. When the methylene chloride was mixed with the acetone, the resultant solvent mixture could dissolve the blend polymers of PLGA and HP55 used to prepare multiple emulsions. The condition of pH 2.0 was used in the preparation of insulin-loaded nanoparticles in order to increase the dissolubility of insulin. The activities of insulin were not reduced much due to the short time of the preparation process. In addition, the nanoparticles were washed three times with DI water and dried, which may also reduce the effect of acid condition on the activities of insulin. These can also be verified by the *in vivo* results followed.

**Figure 2:**
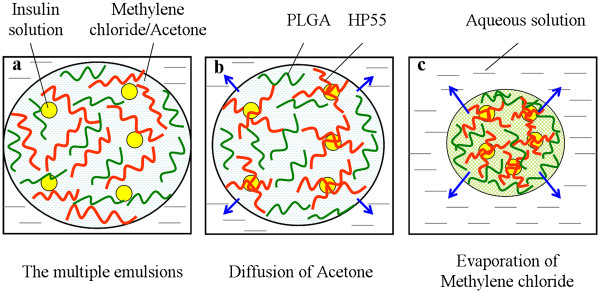
**Phase separation in multiple emulsions.** Includes (**a**) formation of multiple emulsions, (**b**) acetone solvent diffusion and formation of HP55 phase, and (**c**) methylene chloride evaporation and formation of PLGA/HP55 nanoparticles.

Figure 2a shows that insulin solution as the inner aqueous phase could be protected by the oil phase through formation of multiple emulsions. In addition, the improvement of the multiple emulsions may have the chance to uniformly distribute the insulin in the polymer matrix. HP55 phase could firstly be phase separated from the polymer solution in the oil phase after formation of the multiple emulsions, which attribute to the diffusion of acetone from the primary emulsion to the external aqueous solution (Figure 2b). Insulin droplets tend to be distributed in the HP55 matrix via the strong interaction between the carboxylic groups in phthalate groups of HP55 and amide groups in insulin [19]. The formation of PLGA/HP55 nanoparticles occurred after the evaporation of methylene chloride (Figure 2c). The physical stability of PLGA/HP55 nanoparticles will be smartly increased in the acidic environment due to the hydrophobic protection of PLGA and HP55. Hence, the burst release of insulin from PLGA/HP55 nanoparticles in acidic environment would be reduced using the multiple emulsion solvent evaporation method.

**Figure 3:**
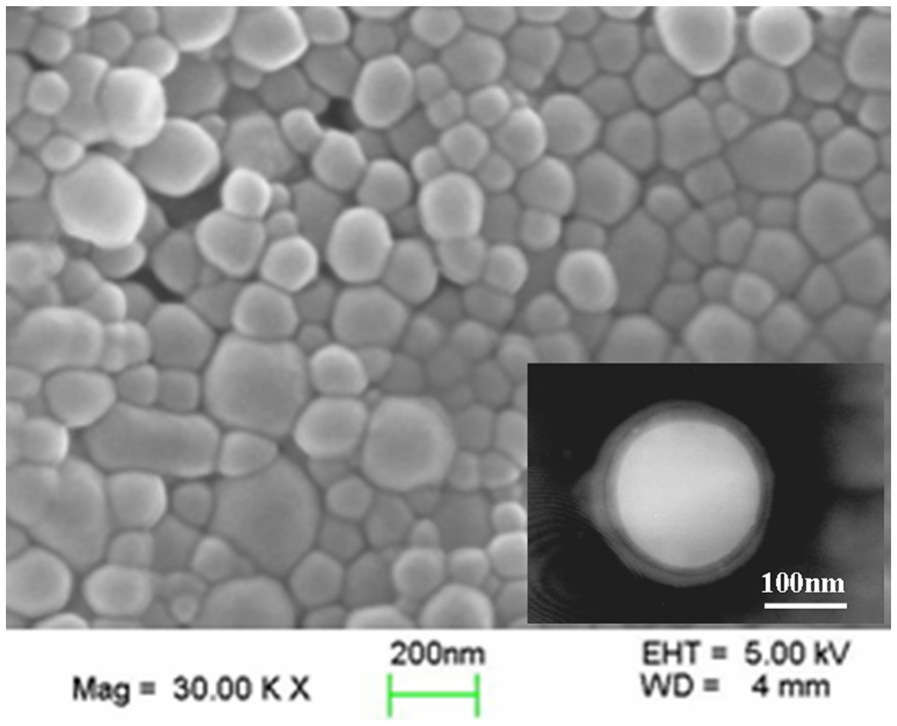
Morphology of insulin-loaded PLGA/HP55 nanoparticles.

The morphologies of PLGA/HP55 nanoparticles by SEM and TEM analyses are shown in Figure 3. The nanoparticles possess a spherical shape and uniform size. The particle sizes of the nanoparticles are listed in Table 1. The results showed that the number of average particle size of the PLGA/HP55 nanoparticles prepared by MESE method was 181.9 ± 19.0 nm (PDI = 0.093 ± 0.031), a little larger than 169 ± 16 nm of PLGA/HP55 nanoparticles prepared by SESD method [19]. In general, the nanoparticles formulated from multiple emulsions were much larger in size than that obtained from simple emulsions [38].

**Table 1:** **Properties of insulin-loaded PLGA/HP55 nanoparticles prepared via MESE and SESD methods (mean ± S.D.,*n*** **= 3)**

	**Particle size (nm)**	**EE (%)**	**LC (%)**
SESD^a^	169 ± 16	65.41 ± 2.3	3.17 ± 0.24
MESE	181.9 ± 19.0	94.25 ± 1.24	5.89 ± 0.17

^a^The data are provided by literature [19]. EE, encapsulation efficiency; LC, loading capacity.

### Drug entrapment ability of PLGA/HP55 nanoparticles

The drug entrapment ability of PLGA/HP55 nanoparticles prepared by SESD and MESE techniques is compared in Table 1. Entrapment efficiency (EE) was significantly dependent on the preparation method [39]. The reason for this lower EE (65.41% ± 2.3%) by SESD is due to the low affinity between the polymer and hydrophilic drug, leading to the drug substance tendency to move from the organic phase to the outer aqueous phase during the single emulsion diffusion process. In order to avoid this, the hydrophilic insulin partitioned between the two immiscible phases (the inner aqueous phase and the oil phase), allowing the drug to be initially dissolved in an inner aqueous phase. Hence, the high EE (94.25% ± 1.24%) was obtained by MESE technique in the study.

The novel MESE preparation technique described here for the encapsulation of hydrophilic macromolecule in PLGA/HP55 nanoparticles resulted in improved encapsulation efficiency in comparison to the SESD technique. Obviously, the success of this technique hinges upon the ability to construct biocompatible nanoparticles that allow high loading of insulin molecules without premature release before reaching the destination, i.e., intestine.

### *In vitro* release profiles from insulin-loaded PLGA/HP55 nanoparticles

**Figure 4:**
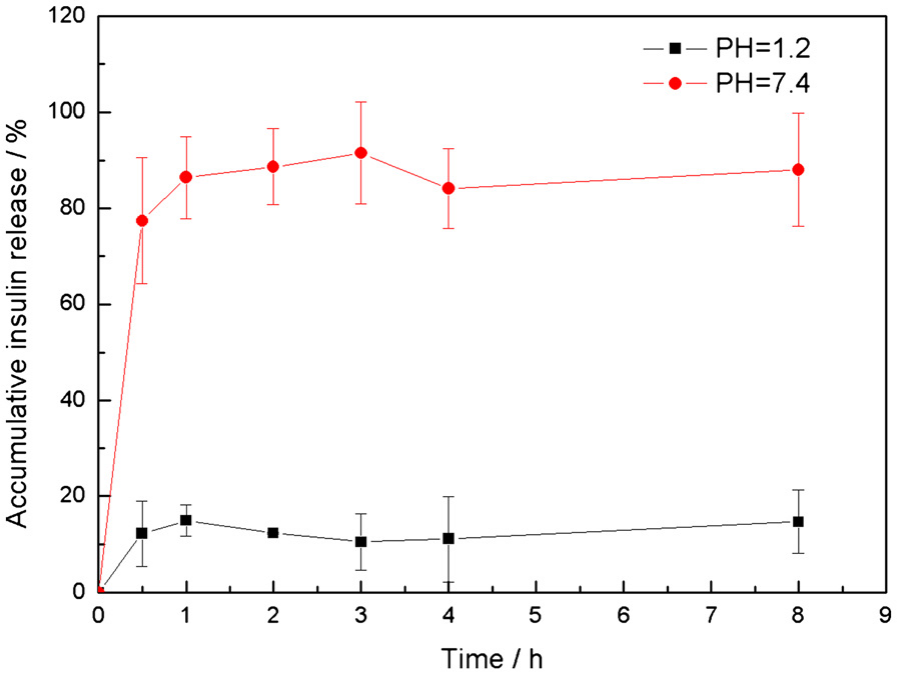
***In vitro *release profiles of insulin-loaded PLGA/HP55 nanoparticles (mean ± S.D.,*n*** **= 3).**

*In vitro* insulin releases of the nanoparticles were performed respectively under gastric environment (pH 1.2) and intestinal conditions (pH 7.4), and the results are shown in Figure 4. For the nanoparticles prepared with pH-sensitive polymer HP55, the release rate of insulin from the nanoparticles is markedly influenced by pH values. At pH 1.2, less than 15% insulin could be released, while at a higher pH value of 7.4, about 90% insulin could be released from the nanoparticles. The sensitivity of insulin release towards pH could mainly depend on the solubility of HP55. At pH 1.2, HP55 and PLGA molecules are hydrophobic ones which could reduce the contact of water and insulin molecules and prevent the release of insulin molecules from the nanoparticles. At pH 7.4, HP55 can dissolve in water, facilitating the release of insulin molecules which mainly distribute in the PLGA/HP55 matrix. The PLGA increases the matrix stability, avoiding the rapid disintegration of nanoparticles, due to its indissolubility in water. Such pH-sensitive release property would be favorable for the protection of orally delivered insulin when nanoparticles passed through the acid environment of the stomach [7,16].

### Oral administration of insulin-loaded PLGA/HP55 nanoparticles in diabetic rats

**Figure 5:**
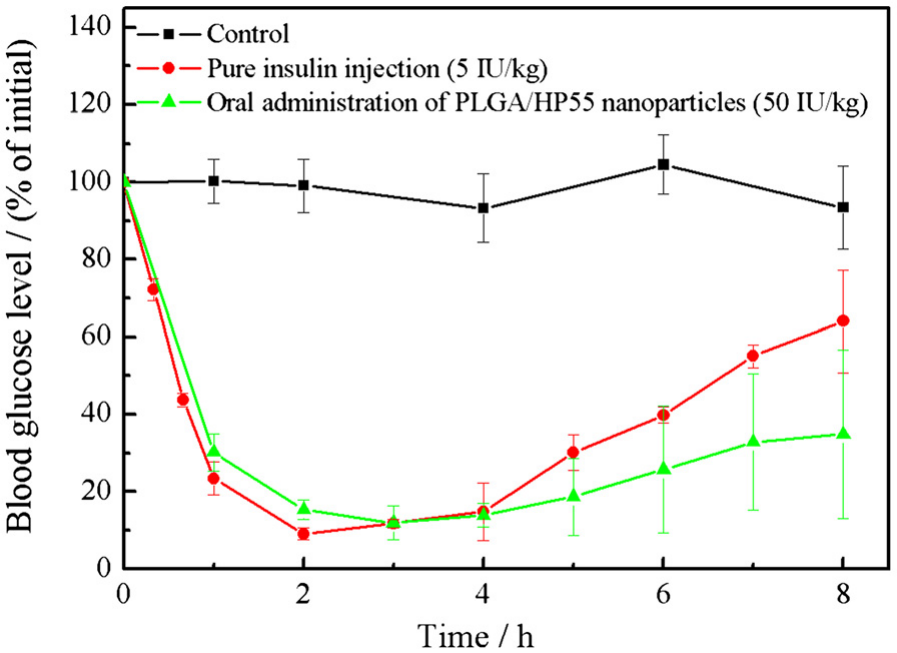
**Plasma glucose levels.** After oral administration of the insulin-loaded PLGA/HP55 nanoparticles (50 IU/kg), blank nanoparticles, and SC injection of insulin solution (5.0 IU/kg) in diabetic rats (mean ± S.D., *n* = 3). Significant difference from blank nanoparticles control (*p* < 0.05).

The pharmacological effect of insulin-loaded PLGA/HP55 nanoparticles was evaluated in diabetic rats dosed orally at loading level of 50 IU/kg; the number of animals per group is three. The changes in plasma glucose compared to insulin injected subcutaneously at 5 IU/kg and blank nanoparticles dosed orally are shown in Figure 5. A significant difference in plasma glucose reduction between insulin-loaded and blank nanoparticles was observed, especially 1 to 8 h after oral administration (*p* < 0.05).

Oral administration of the insulin-loaded PLGA/HP55 nanoparticles and SC injection of the free-form insulin solution produced a significant hypoglycemic effect, suggesting that the preparation and freeze-drying processes did not lead to the fragmentation of insulin molecules. SC injection of the insulin solution produced a sharp decrease in blood glucose levels (90% in 2 h), which gradually returned to the basal levels after 8 h. The fast and prolonged reduction in blood glucose levels was investigated between 1 and 8 h after oral administration of the nanoparticles. This phenomenon of fast reduction of blood glucose levels was also conformed within 2 h after oral delivered aspart-insulin or insulin-loaded nanoparticles, which could be attributed to the initial release of insulin from the nanoparticles [24,28]. The quick pass of nanoparticle suspension through the stomach of fasted rats also has the promoted effect on the rapid reduction of blood glucose. The advantages of the present pH-sensitive nanoparticles are the use of a multiple emulsion method increasing the drug entrapment ability and addition of HP55 that greatly increase pH-sensitivity of nanoparticles. Compared to the nanoparticles developed by Cui et al. [19], showing similar PLGA/HP55-based nanoparticles, the present nanoparticles showed an improved entrapment ability by increasing the encapsulation efficacy by 1.4-fold and improved release property by increasing the release of insulin from nanoparticles at pH = 7.4 within 1 h by 1.3-fold. These improvements would promote the insulin absorbed as a free peptide in the upper intestine either by a paracellular pathway or a receptor-mediated pathway.

Insulin could be directly absorbed through the intestinal cell exerting a hypoglycemic effect [31,32]. The direct uptake of insulin has been attributed to specific insulin receptors in intestinal enterocytes and rapid internalization of the nanoparticles by the epithelial cells [23,40,41]. The upper intestinal area seems to be the most active region for insulin absorption, improved under fasting conditions [35]. Hence, the fast reduction of blood glucose level after oral insulin-loaded PLGA/HP55 nanoparticles could be attributed to the fast release of insulin in the upper intestine, wherein the pH is approximately 6.0 to 7.0 [42].

**Figure 6:**
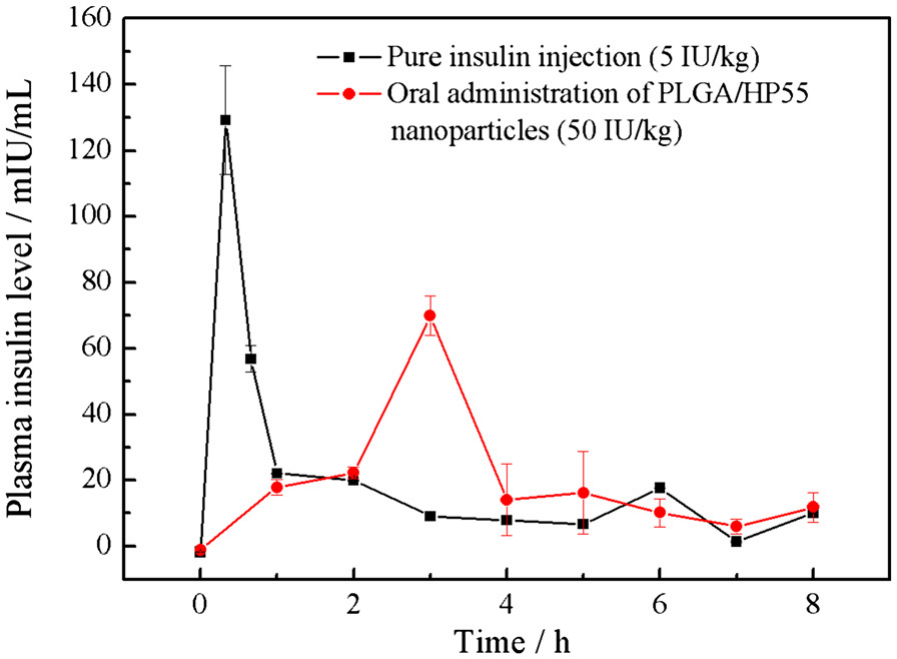
**Serum insulin levels.** After oral administration of insulin-loaded PLGA/HP55 nanoparticles (50 IU/kg) and SC injection of insulin solution (5.0 IU/kg) in diabetic rats (mean ± S.D., *n* = 3).

The intestinal absorption of nanoencapsulated insulin was evaluated by measuring serum insulin levels in diabetic rats. Corresponding plasma insulin concentration-time profiles and the related pharmacokinetic parameters are shown in Figure 6 and Table 2, respectively. As shown, insulin levels increased 1 h after oral administration of insulin-loaded PLGA/HP55 nanoparticles, and the one insulin peak was observed at 3-h post-administration. The initial burst seems to be of value regarding the mechanism of absorption of insulin [28]. The only one insulin peak at 3 h is probably related with the insulin initially released from PLGA/HP55 nanoparticles allowing a high drug gradient concentration directly absorbed through the receptor-mediated pathway or paracellular pathway [29,40,41]. The AUC (0 to 8 h) for the group orally treated with the insulin-loaded PLGA/HP55 nanoparticles was 161.69 ± 28.58 μIU h/mL, which corresponded to a bioavailability of 11.3% ± 1.05% showing improved insulin absorption.

**Table 2:** Pharmacokinetic parameters of insulin in diabetic rats

	**SC-administered insulin solution**	**Orally administered insulin-loaded nanoparticles**
Dose (IU/kg)	5.0	50
*C*_max_ (μIU/mL)	129.21 ± 8.49	69.93 ± 7.64
*T*_max_ (h)	0.33	3
AUC_(0 to 8 h)_ (μIU h/mL)	143.76 ± 21.37	161.69 ± 28.58
BA_R_ (%)	100	11.3 ± 1.05

Parameters after SC administration of the free-form insulin solution or oral administration of insulin-loaded PLGA/HP55 nanoparticles. AUC, area under the plasma concentration-time curve; BA_R_, relative bioavailability; *C*_max_, maximum plasma concentration; *T*_max_, time at which *C*_max_ is attained. Data are given as mean ± S.D. (*n* = 3).

The bioavailability for insulin-loaded PLGA/HP55 nanoparticles obtained by the SESD technique is 6.27% ± 0.42%. By introducing the MESE method into the preparation of PLGA/HP55 nanoparticles, the physiological effect was improved by increasing the bioavailability by 1.8-fold. Compared with PLGA/HP55 nanoparticles prepared by SESD technique, the encapsulated insulin by MESE technique can be released very rapidly allowing a high drug gradient concentration in the upper intestine followed by a good absorption.

## Conclusions

In this study, novel PLGA/HP55 nanoparticles with a pH-sensitive characteristic were prepared by MESE technique for oral delivery of insulin. The PLGA/HP55 nanoparticles exhibited an excellent insulin entrapment ability and a good pH-sensitive release behavior. Additionally, the pharmacodynamic and pharmacokinetic evaluations of orally administered PLGA/HP55 nanoparticles in diabetic rats indicated that absorption of insulin in the upper intestine was fast, and the hypoglycemic effect was significant. These results suggested that the PLGA/HP55 nanoparticles developed in the study might be employed as a potential approach for the multiple daily oral delivery of insulin.

## Competing interests

The authors declare that they have no competing interests.

## Authors’ contributions

ZMW and LL carried out all the experiments and drafted the manuscript. LYZ carried out the *in vivo* study and contributed to the revision of the manuscript. WJ and KQL participated in the *in vivo* study. XDG, YQ, and LJZ received the study, guided its design, the interpretation of the results, and revision of the manuscript. All authors read and approved the final manuscript.

## Authors’ information

ZMW is a doctoral candidate, LL and WJ are master students, XDG is an assistant professor, and YQ and LJZ are professors in the School of Chemistry and Chemical Engineering, South China University of Technology, Guangzhou, People’s Republic of China. LYZ is a research assistant in the Department of Chemical and Bio-molecular Engineering, School of Engineering, The Hong Kong University of Science and Technology, Hong Kong. KQL is an associate professor in the Division of Bioengineering, School of Chemical and Biomedical Engineering, Nanyang Technological University, Singapore.
